# Malarial pathocoenosis: beneficial and deleterious interactions between malaria and other human diseases

**DOI:** 10.3389/fphys.2014.00441

**Published:** 2014-11-21

**Authors:** Eric Faure

**Affiliations:** Aix-Marseille Université, Centre National de la Recherche Scientifique, Centrale Marseille, I2M, UMR 7373Marseille, France

**Keywords:** malaria, pathocoenosis, comorbidity, malaria-therapy, syphilis, plague, cholera

## Abstract

In nature, organisms are commonly infected by an assemblage of different parasite species or by genetically distinct parasite strains that interact in complex ways. Linked to co-infections, pathocoenosis, a term proposed by M. Grmek in 1969, refers to a pathological state arising from the interactions of diseases within a population and to the temporal and spatial dynamics of all of the diseases. In the long run, malaria was certainly one of the most important component of past pathocoenoses. Today this disease, which affects hundreds of millions of individuals and results in approximately one million deaths each year, is always highly endemic in over 20% of the world and is thus co-endemic with many other diseases. Therefore, the incidences of co-infections and possible direct and indirect interactions with *Plasmodium* parasites are very high. Both positive and negative interactions between malaria and other diseases caused by parasites belonging to numerous taxa have been described and in some cases, malaria may modify the process of another disease without being affected itself. Interactions include those observed during voluntary malarial infections intended to cure neuro-syphilis or during the enhanced activations of bacterial gastro-intestinal diseases and HIV infections. Complex relationships with multiple effects should also be considered, such as those observed during helminth infections. Moreover, reports dating back over 2000 years suggested that co- and multiple infections have generally deleterious consequences and analyses of historical texts indicated that malaria might exacerbate both plague and cholera, among other diseases. Possible biases affecting the research of etiological agents caused by the protean manifestations of malaria are discussed. A better understanding of the manner by which pathogens, particularly *Plasmodium*, modulate immune responses is particularly important for the diagnosis, cure, and control of diseases in human populations.

## Introduction

More than 1400 parasite species, including viruses, bacteria, fungi, protozoa and helminths, infect humans (Taylor et al., 2001), and the simultaneous presence of multiple species or of multiple strains of the same species (co-infection) in the human body is commonplace (Cox, 2001; Brogden et al., 2005; Balmer and Tanner, 2011). Therefore, it is likely that the true prevalence of co-infection exceeds one sixth of the global population (Griffiths et al., 2011). Because interactions among co-infecting parasites, which are also termed poly- or multi-parasitisms, can affect host pathology, parasite transmission, and virulence evolution, this phenomenon is of great interest to biomedical research (Rigaud et al., 2010); furthermore, it may lead to biases in the analyses of clinical studies because most co-infections possess associated comorbidities.

Historically, malaria was probably one the diseases with the greatest opportunity to interact with other diseases because of the extent of the malarious areas, of the level of endemicity and of the fact that humans could be infected during all the duration of their lives (Sallares, 2005; Faure, 2012, 2014). Moreover, since Hippocrates and Galen, ancient authors, who were generally shrewd observers, highlighted the increased risk of co-infections in individuals with malaria (Sallares, 2002), and it may be particularly interesting to confront their assumptions with the most recently reported molecular data.

Despite progress in malaria management, it continues to impact annually hundreds of millions of people (WHO, 2013) and therefore, the number of potential co-infections involving this parasitosis remains very high. The focus of this article is to underline some aspects of the complexity of the relationships between malaria, other diseases and the human genome.

## Malaria is a protean disease with devastating consequences on human beings

Malaria is an acute and chronic disease caused by obligate intracellular protozoan parasites of the genus *Plasmodium*, transmitted through the bite of female *Anopheles* mosquitoes. Five species of malaria parasites infect humans (Igweh, 2012): *Plasmodium falciparum* causes malignant tertian malaria, *P. vivax* and *P. ovale* (found only in Africa) cause benign tertian malaria, *P. malariae* causes benign quartan disease, and *P. knowlesi* primarily infects non-human primates. Repeated bouts of tertian or quartan fevers are caused by the cyclic release of merozoite parasites from lysed erythrocytes every 2 or 3 days, respectively (more specifically, febrile paroxysms occur every third or fourth day, respectively, counting the day of occurrence as the first day of the cycle) and commonly result in body temperatures as high as 41°C; however, malarial fevers can also be irregular, continuous or low-grade, as in children or during primary infections and co-infections (Igweh, 2012). Moreover, concerning *P. vivax*, the term “benign” tertian malaria is a misnomer, as this species can cause serious and fatal illness with severe clinical syndromes, including comatose cerebral malaria, and acute renal, hepatic and pulmonary dysfunctions (Baird, 2013). *P. vivax* risk should be considered seriously because this species is responsible for approximately 60–80% of all malarial infections worldwide (Leoratti et al., 2012).

Today, malaria is considered as a tropical disease because of its prevalence in warm regions, but historically it was present in all climate zones worldwide; e.g., in Europe, this disease extended widely from the Mediterranean Basin to the coasts of the Arctic Sea (Huldén and Huldén, 2009; Faure, 2012; Faure and Jacquemard, 2014). Malaria was likely the most deadly of all human diseases, and its impact on humanity has been the subject of much speculation. Authors have held that its ravages disrupted the socioeconomic fabric of societies and even led to the decline and disappearance of civilizations (e.g., Jones, 1907; McNeill, 1979).

In several areas of the past Afro–Eurasia (and later America), the levels of malarial endemicity led to the infection of a great number of humans every year, which may have reduced life expectancy (Sallares, 2002; Roucaute et al., 2014). *Plasmodium* infection during pregnancy strongly increases the risk of neonatal mortality, and young children are particularly vulnerable to malaria (Carter and Mendis, 2002; WHO, 2013). Hundreds of gene mutations confering a survival advantage against malaria are known (Patrinos et al., 2004). Considering the most common mutations, more than 15% of the world population bears mutations conferring resistance to malaria (Dean, 2005; WHO, 2006; Cappellini and Fiorelli, 2008). Several of these mutations are under balancing selections, which maintains deleterious alleles in a population at a relatively high frequency (Barton and Keightley, 2002). For example, sickle-cell gene mutations are well-known examples of balancing selection. In this case, the heterozygote has a higher fitness than both the mutant and the normal homozygote. This seems to indicate that *P. falciparum* was so deadly that it is better to risk a 25% probability of a dead child (homozygous for the sickle-cell gene) than to forsake the possibility of a way to fight against the parasite, and shows evidence of the high level of endemicity and continual selection pressure exerted by malaria in the past.

During the 19th century, over half of the world's population was at significant risk of contracting malaria, and out of those individuals directly affected by this disease, at least 1 in 10 could expect to die (Carter and Mendis, 2002) despite the use of quinine in several countries. Malaria is nowadays still the dominant infectious disease in regions of Sub-Saharan Africa and South Asia (WHO, 2013). Despite concerted efforts to reduce its deleterious impacts, the estimated cases and deaths in 2012 were approximately 607 million and 627,000, respectively. Moreover, the number of deaths may be greatly underestimated and could likely be doubled (Murray et al., 2012). Because malaria is endemic in over one third of the world, with 3.3 billion people at risk of infection (WHO, 2013), the risk of deleterious co-infections with other pathogens is high. Some of these pathogens are major overlooked tropical diseases, but the most prevalent co-infections involve HIV/AIDS, tuberculosis and/or helminthiases (Troye-Blomberg and Berzins, 2008).

Malaria is a protean disease: its clinical manifestations and symptoms can be quite diverse and may be similar to other disease entities. Protean clinical manifestations of malaria are known since at least the 17th century (Morton, 1692). Moreover, *Plasmodium* infections can also produce different results with different biocoenoses (Roucaute et al., 2014). This has major implications in the search for the causative agent(s) of the observed clinical manifestations. Single infections can be misdiagnosed, co-infections can be falsely suspected, or, contrarily, one of the organisms involved in a true co-infection may remain unsuspected. For example, severe multiorgan disorders affecting, lung, heart, liver, spleen, kidney, and intestines are associated with *P. falciparum*, as well as with other species such as *P. vivax* (Baird, 2013). Among these manifestations, gastro-intestinal symptoms are predominant, and brain damage and respiratory disorders are common (Sallares, 2002). Many case reports have also been published regarding association of *P. falciparum* malaria and symmetrical peripheral gangrene (Agrawal et al., 2014), which is a symptom that has been observed during typhus epidemics (*Rickettsia prowazekii*). Misdiagnoses with other diseases with similar signs and symptoms are frequent: diseases with non-specific periodic fever such as gastroenteritis, typhoid fever, hepatitis A, influenza, pneumonia or bacterial meningitis have been diagnosed although the implication of malarial infections alone were subsequently validated (e.g., Mehndiratta et al., 2013; Nayak et al., 2013). Misdiagnosed patients frequently responded well to treatment with cinchona (or quinine since the 1820s), evidencing the implication of malaria (Roucaute et al., 2014). Contrarily, in endemic areas, other diseases might also be misdiagnosed as malaria (e.g., Källander et al., 2008).

## In the ancient world, malaria was likely the most important component of pathocoenoses

The concept of pathocoenosis was introduced by Grmek (1969, 1991), who proposed to consider diseases of a given host population as a whole, thereby integrating both historical and geographical dimensions. Grmek defined pathocoenosis as follows: *“By pathocoenosis, I mean the qualitatively and quantitatively defined group of pathological states present in a given population at a given time. The frequency and the distribution of each disease depend not only on endogenous—infectivity, virulence, route of infection, vector—and ecological factors—climate, urbanization, promiscuity—but also on frequency and distribution of all the other diseases within the same population”*. This neologism was modeled from the term “biocoenosis” which was an old ecological concept coined by Karl Mobius. The pathocoenosis concept also fits with Braudel's “longue durée” paradigm (Arrizabalaga, 2005) even though Grmek particularly insisted on the occurrence of ruptures and breaks in pathocoenoses. The introduction of a new pathogen (such as *Plasmodium*) would induce a breakdown in pathocoenosis, causing a shift toward a supposedly novel state of equilibrium. Indeed, pathocoenoses are constantly evolving, but these dynamic states are difficult to comprehend.

Sallares (2005) considered that “where malaria occurred in antiquity, in the long run it was the single most important component of the pathocoenosis, or ecological community of pathogens, not only because of its own direct effects on mortality and morbidity but also because of its synergistic interactions with other diseases, especially respiratory and intestinal diseases. This combination drastically reduced both life expectancy at birth and adult life expectancy in areas where malaria was endemic.” While this is not absolute proof that malaria was the most important component of past pathocoenoses (including not only those of the classical antiquity), strong arguments exist in support of this hypothesis, suggesting that malaria was the most frequent fatal disease due to both direct and indirect effects. Several indirect empirical studies support the Grmek's hypothesis suggesting that the prevalence of a disease could depend on that of “all other diseases.” Indeed, in numerous well-documented cases, effective malaria-specific interventions (e.g., insecticide spraying, insecticide-impregnated bed-net programs or implementation of preventive treatments) have lead to reductions in mortality several-fold greater than would have been expected on the basis of the estimation of malaria-related mortality (e.g., Shanks et al., 2008; Dicko et al., 2012). For example, in Guyana, in the 1940–50s, the number of deaths from all causes (including non-vector transmitted diseases) decreased with deaths from malaria and followed the elimination of the mosquito vector (Giglioli, 1972). Similarly, in an Italian town in 1925, all of the children had splenomegaly (enlarged spleen often associated with chronic malaria), but only a dozen individuals had symptoms of acute malaria, and only 8% of all deaths were directly attributed to malaria. After the eradication of malaria in this town, the crude death rate fell from 41 per 1000 to 20 per 1000 (Sallares, 2002). It is difficult to quantify the direct and indirect contributions of malaria to overall mortality, and the interdependence between various diseases, but the evidences suggest it is highly significant (see Shanks et al., 2008).

The impacts of infectious agents not only depend on their associated morbidity but also on the general health of the affected population. Many epidemics due to malaria or other diseases have coincided with periods of famine, affecting underprivileged populations (Nájera et al., 1998). Recent literature on the relationship between malaria and nutrition is controversial; however, nutritional deficiencies are frequent in malaria-endemic areas, and vitamin intake may play an important role in the proliferation of the malaria parasite (Nankabirwa et al., 2010; Javeed et al., 2011). Even if malnutrition and malarial impacts are not directly linked, their inter-relationship is generally accepted as being synergistic, with one promoting the other, and a similar synergism is likely to occur in the development of other diseases (Nájera et al., 1998). Thus, the consideration of socio-economic determinants likely does not compromise the analyses of the major implications of malaria in past pathocoenoses.

The deleterious effects of malaria during co-infections were primarily hypothesized by Hippocrates (Sallares, 2002) and have been empirically theorized by physicians during the 18-19th centuries, principally in France and England (Dobson, 1997; Roucaute et al., 2014). The physicians' observations suggested frequently that malaria was present simultaneously with other diseases: (1) one disease could replace another, e.g., during epidemic peaks, a new disease (such as cholera) could seem to temporarily replace malaria; (2) most diseases, if they persisted, took on a periodic form; (3) periodic nature of fevers could accompany non-epidemic diseases; (4) repeated bouts of malaria might render individuals more vulnerable to other diseases; and (5) in malaria-free cities, the health situation was particularly good and the frequencies of other diseases and epidemics were relatively low. Current knowledge provides scientific explanations for these empirical observations; indeed, besides the high rate of malarial endemicity, there is strong evidence that malaria can lead to an altered immune response.

## The very complex relationships between immunity and malaria

During a co-infection, if one of the two pathogens perturbates the immune response, the consequences for the host might be very severe. Numerous studies have shown that malaria profoundly affects the host immune system by mediating both immunosuppression and immune hyper-activation (e.g., Chêne et al., 2011; Butler et al., 2013; Pradhan and Ghosh, 2013). *P. falciparum* malaria disturbs, among other immune functions, the specific T-cell response, the induction of regulatory T cells and cytokine balance and is associated with polyclonal hyper-immunoglobulinaemia, production of multiple autoantibodies and suppression of specific antibody responses (Cunnington, 2012). A deregulated balance of the host immune response has also been observed during *P. vivax* infection (Leoratti et al., 2012; Rodrigues-da-Silva et al., 2014). By their distribution, burden of disease and impact on immune responses, *P. falciparum* and to a lesser extent *P. vivax* are the two *Plasmodium* species that have the greatest negative effect on human populations (Leoratti et al., 2012; Maguire and Baird, 2014). Although severe illness also occurs with *P. malariae* and *P. ovale*, these seem to be relatively rare events.

Malaria-induced hemolysis impairs also the immune response via, among other mechanisms, hemozoin produced during heme detoxification. Hemozoin is a strong modulator of the innate immune response, and has the potential to be detrimental or beneficial to the host, most likely depending on the stage of the infection (Cunnington, 2012; Olivier et al., 2014). Moreover, malaria infection results in alterations in splenic structure, which may affect the qualities of antibody responses and may impact the development of immunity to other infections (reviewed by Del Portillo et al., 2012). To date, data to determine whether malaria-induced immunosuppression (“immunomodulation” may be more appropriate; Cunnington and Riley, 2010) is significant in the long-term are lacking. Moreover, although acute malaria infection may impair the immune response, the consequences of immunological changes due to persistent immune activation during recurrent or persistent infections are poorly known; however, both asymptomatic and symptomatic *P. falciparum* malarial infections suppress immune responses to heterologous polysaccharides and sometime protein antigens in vaccines (Cunnington, 2012). Additionally, antimalarial treatment may enhance responses to some types of vaccines (Cunnington and Riley, 2010). While it is still unclear how parasite-induced changes in the host immune response influence the clinical manifestations of *Plasmodium* infections, malaria is associated with poor antibody responses to chronic Epstein-Barr viremia and an increase in HIV viral load and bacteremia (Cunnington and Riley, 2012).

## Clinical importance of malaria co-infections with other diseases

### Brief terminology of disease interactions

Humans, like other metazoans, are continuously infected by a very wide variety of agents that may disappear without the development of clinical signs (Girard et al., 1998). Similarly, co-infection does not necessarily imply a morbid state, and co-infection and comorbidity are non-synonymous occurrences. In this article, the term co-infection refers to two or more species or strains of parasites that are simultaneously present in a given host, regardless of the presence of morbid signs. When in doubt, the use of this word is preferred over that of comorbidity. This latter term is used in cases in which two or more infectious diseases co-occur simultaneously in the same individual, indicating that each infectious agent has caused the clinically evident impairment of normal functioning.

Infectious agents can interact in various ways (both directly and indirectly) with each other and with their host. With regard to ecology in general and the microbial world in particular, one population can influence a second one in different ways, and these effects can be positive, negative or neutral (e.g., Wilson, 2009). However, true neutrality is virtually impossible to prove, and may be wrongly presumed due to limited knowledge of the relationships between pathogens. Cooperative or interfering interactions are frequently defined as synergistic or antagonistic (Mayhew, 2006). In antagonistic interactions, one infectious agent benefits at the expense of another, but one pathogen can negatively interfere with another without deriving cost or benefit; in this case, the term “amensalism” is more appropriate. Synergism is a mutually advantageous relationship, commensalism is the term used when only one of the partners benefits from the other without either harming or benefiting the latter. The direct interaction of an infectious agent with another can occur within the same host; but indirect effects mediated by the host seem to explain most cases of disease interactions, which principally occur via the immune system. Thus, the terms “interactions between infectious agents” and “interactions between infectious diseases” are not equivalent. During disease co-occurrence, neutral (two or more comorbid diseases with no apparent interactions), synergistic and antagonistic combinations of diseases have been observed (Gonzalez et al., 2010). However, co-infections may result in overall interactions between conditions that are not necessarily strictly neutral, synergistic or antagonistic, but that likely represent a continuum of interactions similar to what occurs between microorganisms. Because of the lack of specific terms available for describing some types of disease interactions, the interactions between malaria and other diseases have been divided into two large groups; those for which the co-infection negatively affects the other disease, and those for which the co-infection positively affects the other disease or results in a synergistic interaction.

### Examples of true or supposed negative interactions

#### Malarial fever therapy applied to the treatment of neurological syphilis

A well-known example of negative interaction between pathogens is that between those responsible of malaria and syphilis. From the early 1920s until the advent of penicillin in the mid 1940s, a therapeutic strategy using malarial inoculation in the treatment of syphilitic patients with dementia paralytica was used in Europe and North America (reviewed by Snounou and Pérignon, 2013). The Austrian neuro-psychiatrist Julius Wagner Jauregg (Nobel Prize in 1927) was considered the initiator of this method. The inoculation (generally of *P. vivax*) was performed using infected blood or more rarely by the bite of infected mosquitoes. Malaria was cured using quinine only after some fits of fever. Some patients were effectively cured of syphilis, and many went into partial remission or observed a halt in the progression of their symptoms. However, the Hippocratic principle *primum non nocere* was not pre-eminent; e.g., some patients with syphilis who were treated using *P. vivax* subsequently died, and higher mortality rates (up to 15%) were observed in individuals with additional comorbidities (Baird, 2013). The so-called “malaria-therapy” has greatly contributed to our knowledge of human malarial infections but our understanding of the effectiveness of this treatment is recent. The *in vivo* optimal temperature range of *Treponema pallidum* is 33–35°C, rendering this bacterium sensitive to malaria-induced fever (Snounou and Pérignon, 2013). However, in co-infected individuals, syphilis can be more rapid in its course and resistant to treatment in proportion to the chronicity of the malarial infection; moreover, malarial infection might arouse latent syphilis (Deaderick, 1909). Molecular studies have revealed that this could be due to synergistic effects inducing CD4^+^ suppression in individuals with both malaria and syphilis (Wiwanitkit, 2007). Experimental studies and observations from around a century ago suggest that malarial fevers accelerate recovery from another venereal disease, the gonorrhea, caused by the bacterium *Neisseria gonorrhoeae*, which is also very sensitive to temperature; a temperature increase of only a few degrees kills these pathogens (Anonymous, 1929; Simpson, 1936).

#### Malaria and tuberculosis

Until the 19th century, it was believed that tuberculosis (pulmonary phthisis) was rare or absent in highly malarial endemic areas because of an antagonism between tuberculosis and intermittent fevers (Bidlot, 1868). However, more recent data contradict this hypothesis. Experiments using murine *in vitro* and *in vivo* models have demonstrated a bi-directional effect: malaria exacerbates *Mycobacterium tuberculosis* infections, whereas this pathogen modulates the host response to malaria (Hawkes et al., 2010; reviewed by Li and Zhou, 2013). Immune dysregulation, lung pathology and hemozoin loading of macrophages in the context of acute malarial infection led to increased mycobacterial loads (Mueller et al., 2012).

#### Malaria and helminthiases

Helminthiases affect over 800 million people worldwide (Griffiths et al., 2014), and there is significant geographical overlap between these parasites and *Plasmodium* and co-infections are numerous: malaria co-occurs with 80–90% of all soil-transmitted helminth cases worldwide (WHO, 2011). To date, there is still no clear picture of the impact of intestinal worms on malaria and *vice versa* (Adegnika and Kremsner, 2012; McSorley and Maizels, 2012). *Ascaris* and platyhelminths may be protective against malarial infection and severe manifestations, whereas hookworms seem to increase malarial incidence (Nacher, 2011; Lemaitre et al., 2014). Interestingly, in murine models of malaria-helminth co-infection, cross-reactive antibody responses have been observed, in which antigens from both pathogens are recognized. Moreover, co-infection with *Plasmodium* and schistosome parasites, which are phylogenetically distinct from helminths, leads to similar results. These cross-reactivities may be relevant in other co-infection systems and warrant further attention because of their potential influences on disease outcomes (Fairlie-Clarke et al., 2010 and references therein). True synchronous co-infections are relatively rare, they may result from the ingestion of contaminated food or drinking water or tick bites and principally concern bacterial and viral infections (Lantos and Wormser, 2014). With regard to malaria, the mosquito-borne parasitic nematode *Wuchereria bancrofti*, which causes lymphatic filariasis, can be transmitted by the common *Anopheles* malaria vector species in many tropical regions. Thus, this disease and malaria may not occur independently, and the risk of co-infection (excluding the potential risk for co-infectious complications resulting from blood transfusions) may be considerable (Manguin et al., 2010).

#### Malaria and hepatitis B virus (HBV) infection

Several malaria-endemic areas are also highly endemic for HBV infection (Andrade et al., 2011). Furthermore, *Plasmodium* and HBV may utilize common receptors during hepatocyte invasion, suggesting possible interactions at both immunological and cellular levels during co-infections (Freimanis et al., 2012). However, currently, there is no clear consensus regarding whether *Plasmodium* affects HBV infection and *vice versa*. Studies have found conflicting results: each infection appears to evolve independently, but the cross-reactive immune response affects both pathogens. Acute *P. falciparum* malaria can modulate viremia in patients with chronic HBV infection, whereas the latter can diminish the intensity of malarial infection (Freimanis et al., 2012). It is likely that the type of interaction would depend on the HBV genotype, the *Plasmodium* strain or species and the immunological response to malarial and viral infections (from asymptomatic to acute).

### Positive and synergistic interactions

Negative interactions between diseases appear to be rare; much more frequently, pathogens take advantage of an immune system that has been compromised by other infections such as AIDS (Gonzalez et al., 2010).

#### Relationships with virus

***HIV***. According to a WHO report, there were 35.3 million individuals living with HIV infections in 2012, and in the same year, 1.6 million individuals died of AIDS (UNAIDS, 2013). Moreover, as millions of HIV-infected individuals live in malaria-endemic areas, co-infections are frequent (Cunnington, 2012). Several studies have suggested that people infected with HIV have more frequent and more severe episodes of malaria and *vice versa*. HIV and *Plasmodium* both interact with the host's immune system, resulting in a complex activation of immune cells leading to dysfunctional levels of antibody and cytokine production (Flateau et al., 2011; Cunnington and Riley, 2012; Alemu et al., 2013; Berg et al., 2014; Van Geertruyden, 2014). On the one hand, malaria has been reported to increase HIV replication *in vitro* and *in vivo*, and studies using murine models suggest that malarial infection enhances the sexual acquisition of HIV due to, among other factors, the upregulation of HIV co-receptor expression (Chege et al., 2014). On the other hand, HIV infects and destroys CD4^+^ T helper cells, which are responsible for the specific immune response. As the infection progresses, patients become more vulnerable to other infections, including malaria. HIV-positive individuals suffer higher malarial parasitemia and worse clinical outcomes. Patients with HIV and malaria co-infection had significantly more frequent respiratory distress, liver and renal failure than patients with malaria alone (Berg et al., 2014); however, the mechanisms underlying these disease interactions remain unclear and require further investigation (e.g., HIV infection particularly increases the incidence and severity of malarial infection in areas of low malarial transmission Frosch and John, 2012). Moreover, the combinations of antiretroviral and antimalarial therapies could have possible synergistic or antagonistic effects on treatment efficacies and toxicities (Van Geertruyden, 2014). For example, the use of anti-malarial therapy for patients with HIV co-infection is less effective compared to its use in patients with only malaria. Interestingly, anti-HIV drugs have known activities against parasites during the liver stage and asexual blood stage (Hobbs et al., 2013).

***Herpesvirus***. Epstein–Barr virus (EBV) is a gamma-herpesvirus. Epstein–Barr virus infection generally has a benign clinical course; however, this agent is linked to several B-cell (principal target cell) malignancies, including endemic Burkitt's lymphoma (eBL), especially in immunosuppressed hosts (Chêne et al., 2011). Endemic-BL, which is one of the most prevalent pediatric cancers in equatorial Africa and Papua New Guinea, occurs at high incidence in populations where *P. falciparum* malaria is holoendemic (Chattopadhyay et al., 2013; Mulama et al., 2014), and anti-malarial treatment in a holoendemic area has been linked to a decrease in eBL incidence (Chêne et al., 2011). Although the causal association between EBV and eBL has been established, the precise mechanisms of *P. falciparum*'*s* involvement in lymphomagenesis remain unclear. Malarial infection has been associated with both immunosuppression and immunoactivation, leading to B-cell proliferation and an increase in peripheral-blood EBV loads (references in Mulama et al., 2014); however, the incidence of eBL is low relative to the high prevalence of both malaria and EBV within the pediatric populations, suggesting the involvement of additional factors in its etiology. This could be correlated to the following observations: long-term malarial infection may be protective (Chêne et al., 2011) and sickle-cell trait does not confer protection against eBL (Mulama et al., 2014).

Epstein-Barr virus is one of the eight human herpesviruses known to date. These viruses are widely distributed. In immunocompetent hosts, the clinical outcomes are generally benign, whereas in immunodeficient individuals, primary infection or viral reactivation can lead to severe diseases such as meningitis, encephalitis and pneumonitis and to the development of malignancies (Chêne et al., 2011). The replication of some but not all herpesvirus species may also be favored by episodes of malaria (Chêne et al., 2011). Acute *P. falciparum* malarial infection may induce herpes simplex labialis and varicella-zoster virus reactivation (Chêne et al., 2011 and references therein). Moreover, recently, a syndemic relationship between Kaposi's sarcoma (KS) and *P. falciparum* malaria has been hypothesized (Conant et al., 2013) based, among other factors, on the co-incidence of aggressive forms of KS in malaria-endemic regions. Kaposi-sarcoma-associated herpesvirus is necessary but not sufficient to cause KS, and this sarcoma is more common in HIV-co-infected people who have developed immunodeficiency. However, during latency, gamma-herpesvirus may protect mice against malaria, whereas during acute infection, both the morbidity and mortality of malarial infection were significantly enhanced (Haque et al., 2004). It has also been speculated that through the down-regulation of the cytokine cascade, EBV might reduce the most severe effects of malaria in children in Africa (Watier et al., 1993).

***Flaviviruses***. Thanks to vaccinations against yellow-fever virus, co-infections with this virus and *Plasmodium*, which lead to severe health consequences, have virtually disappeared (Wade et al., 2010). Today, dengue fever (also caused by a flavivirus) and malaria are the two most common arthropod-borne diseases and are widespread in American and Asian intertropical regions, where their endemic areas greatly overlap; however, co-infections are considered to be relatively rare (Yong et al., 2012). Conflicting reports exist on whether dengue-malaria co-infection is more severe than either infection alone (Yong et al., 2012), and malaria and dengue infections share many similar clinical manifestations, which may delay diagnosis and may thus be fatal for co-infected patients (Assir et al., 2014).

***Viruses causing “winter respiratory diseases.”***. Some symptoms are similar for malaria and “winter respiratory diseases,” such as pneumonia and influenza, and malaria deleteriously interacts with these diseases (Sallares, 2002; Hawkes, 2012 for pulmonary manifestations of malaria). For example, in several areas during the period 1918–1920, the “Spanish” influenza pandemic more severely affected malaria-infected individuals and, nowadays, co-infections with influenza are frequently associated with longer hospitalization times than single infections (Afkhami, 2003; Shanks and White, 2013). However, influenza could not exacerbate *P. vivax* malaria contrarily to *P. falciparum* malaria (Shanks and White, 2013).

***Smallpox virus***. Although the data can be contradictory, relationships between smallpox and malaria are probable and perhaps more complex than expected. Indeed, a century ago, it was noted that in co-infected individuals, plasmodia could disappear from the blood with the onset of the smallpox. Moreover, mortality in these cases was unusually high (Deaderick, 1909).

#### Relationships with bacteria

Data from the pre-antibiotic era suggest that malaria increases the host's susceptibility to invasive bacterial infections, e.g., it was observed that malaria was often associated with these diseases, even in countries where they were rare in healthy individuals. Moreover, in co-infected individuals, cures using quinine were able to spontaneously clear bacterial infections without additional treatment (Sallares, 2002, 2005; Cunnington, 2012). More recently, a study has shown that in Kenyan children, sickle-cell trait was associated with a strong decreased risk of bacteremia, suggesting that approximately two-thirds of bacteremia cases were attributable to the effect of malaria (Scott et al., 2011). Malaria may cause susceptibility to bacteremia through, among other factors, impairment of phagocytic cell function, immunoparesis, complement consumption, or hemolysis (which may cause neutrophil dysfunction) (Berkley et al., 2009; Cunnington, 2012; Chau et al., 2013). The exacerbation of bacterial infections has been observed during acute malaria, but also when the parasitemia density was very low and during severe anemia, which generally occurs following prolonged or recurrent malarial infections (Cunnington, 2012). According to this last author, these observations might be reconciled by postulating that malaria causes susceptibility to bacteria through a delayed mechanism following onset of either parasitemia or symptoms.

***Relationships with bacteria inducing gastro-intestinal disorders***. The geographical and seasonal (generally during the hottest months of the year in temperate areas) correlations between malaria and gastro-intestinal diseases have been mentioned by past physicians (e.g., Aiton, 1832; Marchiafava and Bignami, 1894). Later, it was demonstrated that bacterial enteric pathogens such as *Vibrio cholerae*, *Shigella* spp., and *Salmonella* spp. cause most cases of severe acute diarrhea. Clinical signs frequently excluded malaria as a diagnosis, even if *P. falciparum* and *P. vivax* can cause gastro-intestinal symptoms similar to those of typhoid fever or dysentery (e.g., Sallares, 2002; Singh et al., 2011; Naha et al., 2012). On the other hand, malaria can exacerbate the effects of bacterial gastro-intestinal diseases, while the characteristic intermittent fits of fever may be masked by the continue fever due to bacterial infections. In addition to the mechanisms mentioned above, malaria may critically enhance the susceptibility to intestinal bacteria by increasing gut permeability (Cunnington, 2012).

Associations between typhoidal and/or non-typhoidal salmonelloses and malaria have been reported frequently in patients with severe complications, in which synergistic interactions have been observed (Sallares, 2002; Pradhan, 2011; Bhattacharya et al., 2013; Shanks and White, 2013). Although typhoid fever (caused by *Salmonella enterica* serotype Typhi) and malaria have different etiologies, some of their clinical features overlap, including hepatic, pneumonial, encephalopathic and gastro-intestinal disorders (Smith, 1982). Moreover, attacks caused by *P. falciparum* may take the form of (sub)continuous fevers, which are typical of enteric infections (Pradhan, 2011). In malarial endemic areas, due to the difficulty of differentiating typhoid fever from malaria, physicians of the 19th century used the term “typho-malaria” when either was suspected (Smith, 1982). This could be an argument against the hypothesis of malaria major role in past pathocoenoses.

The high frequency of mutations in the CFTR gene in European populations and individuals of European descent could be an argument against the dominant role of malaria in the past pathocoenoses. Indeed, mutations in this gene (particularly the ΔF508 deletion), which cause cystic fibrosis in homozygotes, could led to a survival advantage following infections of typhoid, but also secretory diarrhea (including cholera) and tuberculosis in heterozygotes (Poolman and Galvani, 2007). However, typhoid fevers predominately occured in South Asia and Sub-Saharan Africa, and, in temperate areas, cases of typhoid fever were usually observed during the summer months, whereas malaria might affect individuals throughout the year due to relapses. Cholera is also not a likely candidate for the selection of the CFTR ΔF508 mutation because it has probably not been present in Europe before the 1830s. Due to the correlation between the CFTR ΔF508 mutation frequency and the geographic and historical incidences of tuberculosis, Poolman and Galvani (2007) suggested that the putative selective agent for the mutation was a *mycobacterium*. Indeed, tuberculosis was a very deadly disease to the first half of the twentieth century. However, in past Europe, most of the population was rural, and their repeated contacts with environmental *mycobacteria* might have provided cross-protection against other *Mycobacterium* spp., including those inducing tuberculosis (Silva and Lowrie, 1994). Hence, the selective agent(s) for the high frequency of CFTR mutations still remain unclear, but does not contradict the hypothesis of malaria major role in past pathocoenoses.

During the 19th century, links were established between cholera and malaria. For example, during the cholera epidemy in Bengal in 1817–1819, it was noted, “*It appeared that the villages in which it raged most extensively were considered by the natives as comparatively unhealthy and obnoxious to fevers of the intermittent type*” (Farr, 1852). Similarly, since the second cholera pandemic (1832–1837), which raged in several areas of Europe and especially in malarial endemic regions, various authors have suggested a relationship between these two diseases (Vanoye, 1854). In Turin (Italy), cholera began in the neighborhoods where intermittent fevers, especially pernicious fevers, dominated almost every year (Berutti et al., 1835). In Arles (France), a physician mentioned that cholera seemed to have replaced the pernicious intermittent fevers that had previously occurred all year at the same time and none had appeared during the epidemic year (Guyon, 1832). In France, the towns or areas where cholera had the most impact were most often those where malaria was endemic (Dubreuil and Rech, 1836; Fourcault, 1850). Even if malaria is not a “true water-borne disease” (such as an infection by bacterially contaminated drinking water), in some areas, a parallel can be drawn between the ecological conditions in which cholera vibrios thrive and those that favor malarial vector expansion. For example, in the 1830s, a physician observed that individuals residing in the regions of towns at the highest altitudes were much healthier than those living in lower-elevation neighborhoods in terms of malarial impacts and cholera epidemics intensities (Roucaute et al., 2014). An additional report highlighted the surprising fact that during the cholera epidemic of 1832 in southern France, laborers who worked in marshy areas did not contract this disease although they were provided with housing accommodations which caused them to be in close daily contact with sick and dying individuals (Guyon, 1832). This may suggest that acquired immunity against malaria may also confer indirect protection against cholera. Another more plausible hypothesis could be that the cholera symptoms were caused by *Plasmodium* (*P. vivax* in this case) and not by *Vibrio*. Indeed, it is also known that cholera symptoms can be caused by *Plasmodium* and not by *Vibrio*, as observed in a malarious area of Italy where autopsies revealed high levels of *P. falciparum* parasites in the blood vessels of the mucous membranes of the stomach and the small intestine, with relatively few parasites in the rest of the body (Sallares, 2002).

***Putative relationships between malaria and plague***. In 1843, Boudin presented a list of arguments showing potential relationships between plague and malaria: (1) plague epidemics are often preceded, followed and even accompanied by intermittent fevers; (2) the highly malarious areas were often those where plague was endemic [ancient authors believed that plague had a miasmatic origin]; (3) the most favorable seasons for plague transmission were those in which malarial fevers were the most numerous and severe; (4) climatic conditions that ended episodes of intermittent fevers had the same effect on plague; (5) drying of marshes in areas where severe intermittent fevers were common also resulted in the disappearance of plague; and (6) the rise in altitude that strongly reduced the number and severity of intermittent fevers had a similar influence on plague. More recently, Sallares (2006) argued “*that it is illuminating to keep diseases such as typhus and malaria constantly in mind when considering the evidence for historical plague epidemics*.”

As for many other diseases, the use of cinchona barks, and later, quinine, helped to cure some patients suffering from bubonic plague (e.g., Chicoyneau and Sénac, 1744). These results suggest a comorbidity involving malaria and further suggest that the attenuation of the latter disease allowed the cure of some sufferers; however, this element must always be considered with caution because the drugs could not be administered at the proper time and counterfeit drugs were common (Roucaute et al., 2014). Most of the mentions of malaria and plague comorbidities concerned the Balkans, Western Asia and Africa (e.g., Prus, 1846); however, intermittent fevers raged in several areas of Europe and might have been prevalent during plague epidemics, even if characteristic fits of fever might have been masked. For example, in London from 1661 to 1664, intermittent fevers “*raged like a plague*.” These fevers were followed by the true plague of 1665 and 1666, suggesting that co-infections were present at least at the beginning of the epidemic (Parkin, 1873). Similarly, in Italy in 1528 and in the area of Marseille, France in 1720–1722, intermittent fevers accompanied plague epidemics (Chicoyneau and Sénac, 1744; Gardane, 1777; Guyon, 1855). Plague was sometimes misdiagnosed as malaria; for example, a physician of the 19th century considered that “*the mildest form of plague resembles intermittent fever so much, that it was almost impossible to distinguish the disease, before the appearance of buboes*” (Parkin, 1873), and even in the late 20th century, at the beginning of the infection, cases of true plague could be misdiagnosed as malaria (Sallares, 2006).

The true plague is caused by *Yersinia pestis*, and analyses of ancient DNA suggest that this bacterium was the causal agent of the three human plague pandemics (during the 6–8th, 14–17th and 19–20th centuries) even if the strains may have varied (Wagner et al., 2014). Discrepancies between at least the second pandemic and both modern bubonic or pneumonic plague outbreaks have been noted, pertaining to, among other factors, symptomatic descriptions, infectivity and lethality, epidemic velocity, and peak seasonality of mortality (Welford and Bossak, 2009). Comorbidities may explain these discrepancies, including the seasonal inversion. In modern epidemics, the peaks are generally observed in winter; in contrast, in medieval Europe, the plague mortality peaks usually occurred from the late summer through early fall (Welford and Bossak, 2009). Reports on the possible relationship between malaria and plague are too numerous to discuss here. I suggest that in many areas, malarial endemicity could explain some of the particular characteristics of past plague epidemics. Epidemics of plague had also severe consequences in malaria-free areas, suggesting that those plague bacilli were particularly pathogenic or that the populations were highly sensitive, due, among other factors, to co-infections with agents with effects analogous to those of *Plasmodium*.

## Discussion

Co-infection is the rule rather than the exception in nature, and has been documented across diverse systems (Rigaud et al., 2010). However, our understanding of the manner by which multiple infections affect their hosts remains limited, and studies of malaria co-infections could be particularly beneficial. Indeed, this disease, which was likely one of the most important component of historical pathocoenoses, is still a predominant cause of morbidity and mortality worldwide. Moreover, given the high prevalence of malaria exposure in endemic zones and the frequent chronic nature of infection, the risks of comorbidities with other endemic chronic and acute diseases are very high. Malaria, exemplifying the concept of the pathocoenosis, can interact with numerous viral, bacterial and eukaryotic taxa across all major pathogen groups (e.g., Cox, 2001; Sallares, 2005; Cunnington, 2012; Gonçalves et al., 2014). Interest in co-infections involving malaria has increased in recent years (reviewed in Cunnington, 2012; Gonçalves et al., 2014). Most studies consider cases in which both diseases seem to be exacerbated, suggesting synergistic interactions, but other types of relationships have been observed. For example, co-infection does not appear to affect the outcome of malarial infection, while the other diseases can be either aggravated or slowed. Despite the abundant evidence that malaria and other diseases interact, the subtlety and complexity of these interactions at the clinical and immunological levels are far from clear. A better understanding of the manner by which multiple pathogens modulate the immune response is particularly needed, and may illuminate novel approaches for the diagnosis, cure and control of diseases in human populations (Graham et al., 2007). The complex cytokine balance during co-infections involving *Plasmodium* has been summarized in a recent review (Gonçalves et al., 2014). Although the diverse direct and indirect mechanisms are not well-understood, *Plasmodium* may substantially modify the immune response and even induce an immunosuppression-like state, which may complicate the clinical outcome of diseases (Scott et al., 2011; Cunnington, 2012; Sandlund et al., 2013; van Santen et al., 2013). Even supposedly benign malaria (e.g., due to *P. vivax*) may be debilitating, and individuals suffering from repeated attacks of malaria may be less able to resist other infections (Dobson, 1997; Sallares, 2002). Conversely, malaria can also have deleterious effects on the survival of some infectious agents, possibly as a result of the immunomodulation of the immune response mediated by *Plasmodium* (Cunnington, 2012), and also due to the bouts of high fever.

Although there are some disagreements, the evidence indicates that the effects of fever are complex but overall beneficial. Studies have demonstrated that the increased temperature during fever assists healing directly (by physico–chemical activities) and indirectly because it leads to the enhancements of several immunological processes (El-Radhi, 2012). Fever exerts an overall adverse effect on the growth of bacteria and the replication of viruses. Temperatures >37°C restrict a wide range of Gram-negative bacteria (Green and Vermeulen, 1994), and frequent administration of antipyretics to patients with bacterial diseases can worsen their illness (El-Radhi, 2012). The elevated temperature prevents the bacteria from synthesizing the protective lipopolysaccharides that are the major component of the outer membrane of Gram-negative bacteria (Green and Vermeulen, 1994). Lipopolysaccharides contribute greatly to the structural integrity of the bacteria and protect the membrane from certain types of chemical attack. Moreover, in the past, malarial fever was the principal form of treatment for neurological syphilis. It is now proven that its effectiveness was principally due to the “physical” increase in temperature (Snounou and Pérignon, 2013). In addition, most viruses cease to replicate at temperatures between 40 and 42°C (El-Radhi, 2012). Almost all infectious diseases can cause fever, but in malarial endemic areas, only malaria frequently induces high fevers that are able to directly and indirectly reduce the deleterious effects of some other infections on a fairly regular basis. In contrast, malarial fever can also exacerbate viral infections; herpesviruses, for example, respond to an increase of temperature with rapid reactivation and active viral gene transcription of latent virus (Grinde, 2013). Moreover, despite the temperature sensitivities of various Gram-negative bacteria species, malaria exacerbates their infections in individuals, suggesting that during malarial disease, the deleterious effects on the immune response far outweigh the salutary effects of fever, e.g., malarial infections are associated with increase of Gram-negative bacteremia (Cunnington and Riley, 2012).

An improved understanding of the interactions between plasmodia, other pathogens and human hosts is necessary, particularly for viral co-infections, as there is a paucity of data, and for malarial and helminthic co-infections, as some anti-inflammatory profiles are associated with protection, while others are not (Frosch and John, 2012). Further studies are also needed to reveal the temporal and life history-associated stage-specific differences in immune responsiveness (Jackson et al., 2011), and to delineate the modulations of the immune response according to the stages of the disease (chronic or acute, and asymptomatic or symptomatic), the levels of superinfection and reinfection and the malarial endemic rate (which may be correlated with premunition level: after repeated bouts of malaria that occur over a relatively short period of time, an individual develops a non-sterilizing immunity that does not prevent parasites from developing and circulating in the blood after a new inoculation but does generally suppress the development of severe clinical symptoms; Russell et al., 1963). Possible cytokine modulations during periods of fits with fever also need to be analyzed. A deeper mechanistic and clinical understanding of the effects of co-infection on immunity, and especially on the immunomodulation induced by *Plasmodium*, would help the development of efficient vaccines.

The possible consequences of co-infections must also greatly depend on the genotypes of human hosts and pathogens. Malaria infections often involve more than one genotype per species (Mobegi et al., 2012), which has also been commonly observed with many other host parasites (Read and Taylor, 2001; Balmer and Tanner, 2011; Alizon et al., 2013). In their review, Balmer and Tanner (2011) discussed previous theoretical and experimental studies suggesting that mixed infections have broad ranges of clinically relevant effects and involve a number of human infectious agents. These effects include alterations in the host immune response and changes in pathogen and disease dynamics caused by interactions between *Plasmodium* strains, many of which can lead to pathogen evolution. Although the cause of infection by multiple distinct strains is considered to be an important epidemiological parameter for malaria (Doolan et al., 2009), it remains a neglected aspect that is just beginning to be considered for other diseases (Balmer and Tanner, 2011). However, the differences between co-infections caused by different strains of the same species or by different species have been recently discussed in a study reporting the difficulties in evaluating multiple infections by similar strains of the same species (Alizon et al., 2013). Considering the genotypes of parasites involved in co-infections may help to reconcile some of the apparent contradictions in the literature. Moreover, most studies have concentrated their efforts on *P. falciparum* or the murine model of malaria, so to date, data concerning other human *Plasmodium* species are either completely lacking or insufficient to allow any firm conclusions (Cunnington and Riley, 2010). Studies of the relationships between *P. vivax* malaria and other diseases are particularly important to this understanding due to the pathogenicity and endemicity of this species (Baird, 2013).

Two or more types of parasite strains or species infecting an individual host may interact directly by physical (as fever) or by chemical means, and indirectly via “bottom-up” processes (e.g., competition for shared host resources) or “top-down” processes (e.g., facilitation or immune-mediated competition) (Haydon et al., 2003). However, these last authors have stressed the difficulty of disentangling the “bottom-up” and “top-down” processes. Recently, Griffiths et al. (2014) have analyzed over 300 published studies to construct a network outlining the manner by which groups of co-infecting parasites tend to interact, suggesting that pairs of parasite species are most likely to interact indirectly through shared resources, rather than through immune responses or other parasites. The majority of malaria infections consist of multiple competing genotypes and/or *Plasmodium* species, and resource-mediated (e.g., red blood cells), immune-mediated, or potentially, interference competition between strains results in the suppressions of parasite densities (Mideo, 2009; Pollitt et al., 2011). However, although red blood cell density has been shown to affect malaria intensity in laboratory mice and in humans, suggesting competition between *Plasmodium* strains and species (Antia et al., 2008), many of the studies mentioned here highlight the role of immune modulation or of fever in governing the interactions between *Plasmodium* and other infectious agents. Moreover, hemotrophic parasites other than plasmodia (such as protozoa belonging to the class piroplasmidea and bacteria, such as *Bartonella* spp. and *Mycoplasma* spp.) relatively rarely affect humans, and thus, direct competition with plasmodia rarely occurs. Parasites causing hemorrhaging and anemia can impact the age profiles of the red blood cells available for plasmodia (negatively or positively). The case of *Plasmodium*/HBV co-infection is particularly interesting because interactions between these two parasites may be both direct, involving competition for the same attachment sites on hepatic cells, and indirect, via the host's immune system, which has antagonistic consequences (Freimanis et al., 2012). As already suggested by Kochin et al. (2010) further studies are required to determine the respective impacts of innate immunity and resource limitation on the control malaria infections.

Despite the very limited medical knowledge available over 2000 years, ancient authors were aware of the often deleterious consequences of co-infections on human populations, and they have also cited examples of the supposed antagonistic interactions between concurrent diseases (Sallares, 2002). Although critical analyses of these earlier works are not easy because of the limited diagnostic tools and the ambiguity of the terms used, the analyses of these early reports may provide very relevant information regarding past pathocoenoses and the evolution of medical thought. In addition, contradictions with current knowledge may lead to the testing of new hypotheses that may produce valuable data; for example, the examination of the putative deleterious relationship between malaria and plague. The exacerbation of Gram-positive bacterial infections by malaria is currently well-known and also involves *Yersinia enterocolitica* (Scott et al., 2011), which belongs to the same genus as the plague agent. There are difficulties in identifying diseases involved in historical pathocoenoses, given the frequent ambiguity or vagueness of descriptions of diseases in historical sources. Various types of pathogens frequently infected individuals, even if one of them could be dominant, and the very wide variety of symptoms make their etiologies difficult to pinpoint. In addition, malaria's role as an important component of the pathocoenoses could make difficult to determinate the nature of causal agents. First, malaria has protean manifestations, and misdiagnosis can be very common. Second, malaria may be incorrectly dismissed as a diagnosis when intermittent fevers did not occur due e.g., to co-infections. Third, the complex patterns of relationships between malaria and other diseases render retrodiagnosis difficult or even impossible. Fortunately, current developments in technology provide retrospectively “proof” thanks to DNA analyses. Indeed, as for other infectious diseases, direct evidence for malaria can come from the detection of specific DNA sequences in the remains. The DNA of *P. falciparum* has been retrieved from both skeletal remains (Sallares and Gomzi, 2001) and the soft tissues of Egyptian mummies (e.g., Hawass et al., 2010). The successful amplification of ancient plasmodia DNA suggests a massive infection, as a low level of parasitemia would likely go undetected after so much time had passed (Sallares et al., 2004). Despite its longer persistence in the human body, the amplification of ancient DNA is more difficult for *P. vivax* than for *P. falciparum* (Pinello, 2008). In the future, the continual improvement of techniques should not only yield estimates of the relative proportions of each parasite species in an area and for a given period but also should indicate the potential virulence of the latter. Moreover, studies of ancient DNA could yield data regarding the different constituents of past pathocoenoses in malaria-endemic regions. Research on plasmodia DNA constitutes an indispensable preliminary to any study on this topic, as in a recent study that revealed malaria (*P. falciparum*) and tuberculosis co-infections in mummies from Ancient Egypt (Lalremruata et al., 2013). In addition, future research should explore the role of the ancient spread of alleles in conferring resistance. The findings would aid our understanding of the interrelations between the human genome and pathogens over recent millennia and could allow us to date the appearances of these mutations.

To conclude, the improved understanding of the prevalence of co-infection is greatly needed, in part because co-infections—including those with malaria—are often associated with worse host health symptoms and higher parasite abundances compared with hosts with single infections, and they are associated with reduced treatment efficacies and increased treatment costs (Griffiths et al., 2014). Moreover, co-infections are concentrated among the impoverished populations living in developing countries where health needs are the greatest (Bonds et al., 2010). In addition, examples such as the malaria-syphilis comorbidity, highlight the fact that the simplistic black-and-white, manichean perception of the parasite world is partially erroneous. Malaria could not be (always) the quintessence of evil.

### Conflict of interest statement

The Guest Associate Editor Anaïs Baudot declares that, despite being affiliated to the same institution as the author, the review process was handled objectively and no conflict of interest exists. The author declares that the research was conducted in the absence of any commercial or financial relationships that could be construed as a potential conflict of interest.
